# Racial representation of psychological services in a London male remand prison

**DOI:** 10.1192/bjo.2021.233

**Published:** 2021-06-18

**Authors:** Josh Covey, Joanne Williams, Radha Kothari, Scott Bartle

**Affiliations:** 1 HMP Wormwood Scrubs; 2 Barnet, Enfield and Haringey Trust

## Abstract

**Aims:**

To investigate whether racial groups are proportionally represented in referrals for trauma, hearing voices, emotional regulation and psychological therapy.

To understand the psychological needs across racial groups in HMP Wormwood Scrubs, the UK's 4th-most diverse prison.

To see if the long-established under-representation of Asian males and over-representation of Mixed males in psychological services in the community is also occuring in the prison system.

**Method:**

Psychological referrals were recieved via the medical notes system (SystmOne), whereby a prisoner's name, age, location, racial group and reason for referral are trasnferred into the psychology referrals database.

773 referrals were made between October 2018 and May 2020. As the prison's population throughout this time period was fluid, the month of December 2019 was used as a reference for the general prison poipulation.

Racial groups were specified using the Office of National Statistics' 5-category classification system (White, Black, Asian, Mixed and Other).

**Result:**

There is a consistent under-representation of Asian males in psychological referrals in relation to their general prison population. Whilst this group makes up 17% of the population of the prison, only 10% of prisoners referred to psychological services identified as Asian.

Those identifying as Mixed are over-represented in trauma referrals and psychological therapy referrals. The prison's mixed population is 7%, whereas 16% of those being referred for these two reasons were from the same racial category.

The proportion of patients who identified as Black, White or Other and were referred for psychology input were found to be representitive of the wider prison population, suggesting no clear over or under-representation.

**Conclusion:**

Trends seen in the community in regards to Asian males being under-represented in psychological services are also evident in one of the UK's most diverse prison populations.

Public health campaigning to reduce stigma and promote help seeking in BAME communities is of vital importance to provide the needed support for those silently dealing with psychological problems.

The two largest racial groups in the prison, White and Black individuals, where found to be proportionally represented in their respective referrals to psychological services.

One key finding was in regards to Mixed race individuals, who comprise 7% of the total prison population but 16% of psychology referrals. As this racial group is one of the fastest-growing in addition to be over-represented in referrals, it is vital to understand how provisions can be put in place to appropriately address the needs of this group.

